# Supplementary material for: Perceptions and Acceptability of a Low Phytate: Iron Molar Ratio Biofortified Bean and Sweet Potato Dish Among Pregnant Women in Rural Uganda
Source: Nutrients. 2025 May 11;17(10):1641. doi: 10.3390/nu17101641 (PMC12114513; doi:10.3390/nu17101641)
Supplement: Supplementary file 1 [file nutrients-17-01641-s001.zip › nutrients-3573264-supplementary.pdf]

Table S1. Description of the six steps for inductive thematic analysis used in the study

Steps	Description
**Familiarization with data**	A verbatim transcription was done. Verbatim transcription is a word-for-word transcription of a recording. The verbatim transcription was later translated from the local language (*Lhukonzo*) into English by three facilitators. The translated recordings were cross-checked by a *Lhukonzo* native speaker and a professional teacher against the English translation, for accuracy
**Generating initial codes**	The translated data was organized (coded) systematically throughout the complete set of data. Data was then summarized pertinent to each code.
**Searching for themes**	The codes were organized into potential themes by gathering all data relevant to each potential theme.
**Reviewing themes**	Checked if the themes worked regarding the coded extracts and the entire data set, to enable generate a thematic 'map' of the analysis.
**Defining and naming themes**	Ongoing analysis from three independent researchers to refine the specifics of each theme, and the overall story the analysis tells, generating clear definitions and names for each theme.
**Producing the report**	Thereafter, a discussion was written for each theme relating to the analysis of the research question.
